# The ASK1-Signalosome regulates p38 MAPK activity in response to levels of endogenous oxidative stress in the *Klotho* mouse models of aging

**DOI:** 10.18632/aging.100194

**Published:** 2010-09-15

**Authors:** C-C Hsieh, Makoto Kuro-o, Kevin P. Rosenblatt, Reynolds Brobey, John Papaconstantinou

**Affiliations:** ^1^ Department of Biochemistry and Molecular Biology, University of Texas Medical Branch, Galveston, TX 77555, USA; ^2^ Department of Pathology, the University of Texas Southwestern Medical Center, Dallas, TX 75390, USA; ^3^ The Brown Foundation Institute of Molecular Medicine, the University of Texas Health Science Center, Houston, TX 77030, USA

**Keywords:** -signalosome, p38, thioredoxin, senescence, aging, Klotho

## Abstract

Reactive oxygen species (ROS) and elevated levels of p38 MAPK activity accelerate physiological aging. This emphasizes the importance of understanding the molecular mechanism(s) that link ROS production to activation of the p38 mediated promotion of aging, longevity, and resistance to oxidative stress. We examinedKlotho^(-/-)^ (elevated ROS) and Klotho overexpressing mice (low ROS and resistance to ROS) to determine whether the ROS-sensitive apoptosis signal-regulating kinase (ASK1)-signalosome → p38 MAPK pathway plays a role in the accelerated aging of Klotho^(-/-)^, and resistance to oxidative stress and extended lifespan in the Klotho overexpressing models. Our results suggest that increased endogenous ROS generated by Klotho^(-/-)^ and resistance to oxidative stress in Klotho overexpression are linked to the regulation of ASK1-signalosome → p38 activity. We propose that (a) the ASK1-signalosome → p38 MAPK pathway is activated by oxidative stress due to ablation of the Klotho gene; (b) increased longevity by Klotho overexpression is linked to to suppression of the ASK1-signalosome-p38 MAPK activity; (c) the ROS-responsive ASK1-signalosome regulates physiological aging via its regulation of p38 MAPK, through a mechanism that balances the levels of inhibitory vs. activating ASK1-signalosomes. We conclude that the Klotho suppressor-of-aging activity is linked to the ASK1-signalsome, a physiological ROS-sensitive signaling center.

## INTRODUCTION

Endogenous ROS are important factors that determine signaling pathway activities controlling the development of aging and longevity phenotypes, and are therefore, a basic cause of the progressive age-associated declines in tissue functions [1-6]. Some biochemical characteristics of aged tissues are con-sequences of their increased pro-oxidant state, and it has been hypothesized that this affects the activities of key signal transduction pathways that regulate these characteristics [7-10]. Although mitochondria have been identified as a major source of age-associated ROS, other significant ROS generating systems include the small GTPases [7-9] and DNA damage [11]. This emphasizes the importance of understanding the mechanism that links ROS-sensitive signaling to the development of characteristics of aging and longevity [12-14]. Thus, the observations that *Klotho*, an aging-suppressor gene, confers resistance to oxidative stress [14,15], decreases levels of urinary 8-oxoG and activates MnSOD [13] in the *Klotho* overexpressing mice supports the hypothesis that these longevity characteristics may be linked to ROS-sensitive signaling processes and resistance to oxidative stress. At the same time our observations that ROS generated by mitochondrial electron transport chain (ETC) dysfunction activates the p38 MAPK pathway, known to promote senescence [15] and aging [10,16], suggests that the elevated oxidative stress caused by *Klotho* ablation may promote senescence signaling pathways targeted by p38 MAPK. Alternatively, the decreased oxidative stress seen in *Klotho* overexpression may attenuate p38 MAPK activity and senescence pathways thereby promoting longevity [7,12,16-21]. These observations raise the question of whether the mechanism that links *Klotho* ablation-activated ROS to enhanced aging (*in vivo*) involves the sustained activation of the p38 MAPK pathway.

The mechanism of regulation of p38 MAPK activity in response to mitochondrial generated ROS involves activation of the ASK1-signalosome, a ROS-sensitive signaling complex composed of inhibitor and activator proteins [22-24]. We demonstrated that this mechanism involves regulation of the level of a reduced thioredoxin-ASK1 complex [(SH)_2_Trx-ASK1], a component of the inhibitory ASK1-signalosome (inASK1-signalosome) that attenuates the ASK1 → p38 MAPK pathway [21,23,25]. In this mechanism reduced thioredoxin [Trx (SH) _2_] interacts with the N-terminal domain of ASK1 thereby serving as a negative regulator of ASK1 and attenuator of p38 MAPK activity [23,25]. The association, therefore, of Trx(SH)_2_ with ASK1 maintains the inASK1-signalosome as an inactive form. The ROS-mediated oxidation of ASK1-bound Trx(SH)_2_ stimulates dissociation of the complex thereby forming the activating ASK1-siganlosome (actASK1-siganlosome) that activates the ASK1 → p38 MAPK pathway [23,25-30].

Our studies have shown that the (SH)_2_Trx-ASK1complex in the inASK1-signalosome is dissociated by mitochondrial-derived ROS, *i.e.,* rotenone (ROT) an inhibitor of ETC complex I (CI); 3-nitropropionic acid (3-NPA) an inhibitor of ETC complex II (CII), and antimycin A (AA) an inhibitor of ETC complex III (CIII), thus activating ASK1 and its downstream substrates (MKK3 and MKK6) and p38 MAPK [21,31]. We propose that the elevated endogenous ROS levels produced by *Klotho* ablation promotes dissociation of the (SH)_2_Trx-ASK1 complex resulting in the persistent formation of the actASK1-signalosome which sustains elevated p38 MAPK activity thereby promoting aging characteristics. Thus, oxidation of the ASK1-bound Trx(SH)_2_ by mitochondrial generated ROS is linked to activation of the actASK1-signalosome [21]. Alternatively, in oxidative stress resistant *Klotho* overexpressing tissues, low levels of ROS favor increased levels of the inASK1-signalosome thereby inhibiting ASK1. Thus, oxidation of Trx(SH)_2_ and its release from ASK1 links multiple cytotoxic stresses to activation of the p38 MAPK and SAPK/JNK stress response pathways [22,25,30,32,33]. In this study we ask whether a similar mechanism occurs in the *Klotho^(-/-)^* and *Klotho* overexpressing models based on our observation that the inASK1-signalosome levels decrease and actASK1-signalosome levels increase in response to mitochondrial generated ROS. Furthermore, we propose that the (SH) _2_Trx-ASK1 complex of the inASK1-signalosome may be part of the molecular mechanism of resistance to oxidative stress in the *Klotho* overexpressing model and that the attenuation of p38 MAPK through this pathway may play a role in increased lifespan [19,31]. Alternatively, the persistent chronic increase in ROS production in *Klotho^(-/-)^* mice may be a factor that sustains the elevated level of actASK1-signalosome → p38 MAPK activity thereby promoting the processes of aging [21].

Using AML-12 hepatocytes and *Ames* mouse dermal fibroblasts in culture we demonstrated that the (SH) _2_Trx-ASK1 complex level is dramatically decreased in response to mitochondrial ROS generated by ROT, 3-NPA and AA [34], and that the level and activity of actASK1-signalosome and kinases of the p38 MAPK pathway are activated [21,31]. Furthermore, the levels of the inhibitory (SH)_2_Trx-ASK1 complex which are decreased in aged C57BL/6 mice are significantly higher in *Ames* dwarf mice at all ages, suggesting that ROS generated by mitochondrial ETC dysfunction can activate p38 MAPK signaling via the dissociation of the (SH)_2_Trx-ASK1complex. Thus, the attenuation of ROS by *Klotho* overexpression is consistent with the attenuation of p38 MAPK via the increased levels of the inASK1-signalosome [21].

Studies with nematodes [35-38], *Drosophila* [39-42], and rodents [43-49] suggest that the molecular processes that regulate aging and longevity may be similar to those that regulate resistance to oxidative stress. The longevity of the *Klotho* overexpressing, *Snell* and *Ames* dwarf mice has been attributed to their decreased levels of endogenous ROS and their resistance to oxidative stress [45,49,50]. This is supported by the observation that fibroblasts derived from these long-lived mice are significantly more resistant to ROS generators such as H_2_O_2_, paraquat, and ETC inhibitors [21,31,48,51,52]. By comparing the *in vivo* levels of the inhibitory (SH)_2_Trx-ASK1 complex in young vs. old male C57BL/6 mice to those in age-matched long-lived *Snell* dwarf mouse livers (*in vivo)* and in *Ames* derived dermal fibroblasts (*in vitro*) we have shown that these inhibitory complex levels are significantly elevated in the dwarf mouse livers and fibroblasts, and that the corresponding activities of the p38 MAPK pathway are significantly down regulated [21,31]. Similar results linking the ROS mediated regulation of p38 MAPK activity to the levels of the (SH)_2_Trx-ASK1 complex have been reported [30,32,53].Thus, the elevated levels of this inhibitory complex which are indicative of the lower levels of endogenous oxidative stress may be a part of the mechanism of resistance to oxidative stress. Our hypothesis is supported by the report that (a) activation of p38 MAPK in ASK1^(-/-)^ embryonic fibroblasts by H_2_O_2_ and TNF is abolished in these ROS resistant cells [30,32] and (b) the survival of *Snell* and *Ames* dwarf dermal fibroblasts is associated with resistance to oxidative stress generated by H_2_O_2_, paraquat, [32,48,51,52] and ETC inhibitors [21,31]. Mechanistically, these studies suggest that the activity of actASK1-signalosome may be required for the sustained activities of p38 MAPK and SAPK/JNK [21,30,32,53].

The *Klotho* overexpressing mouse, a classical model of longevity [54], shares its characteristics of low levels of endogenous oxidative stress with the *Ames* and *Snell* dwarf mice. Their extended lifespans are attributed to their resistance to oxidative stress [14,45,50,55]. We thus proposed that these models showing low endogenous ROS, would also exhibit elevated levels of the inhibitory (SH)_2_Trx-ASK1 complex. Our studies have shown that elevated levels of this complex are an *in vivo* characteristic of tissues from both *Ames* and *Snell* dwarf models. Thus, according to our hypothesis tissues from *Klotho^(-/-)^* mice should exhibit decreased levels of the (SH) _2_Trx-ASK1 complex, indicative of elevated oxidative stress and those from *Klotho* overexpressing mice should exhibit increased levels of the complex and attenuation of the p38 MAPK pathway, indicative of low endogenous ROS. In these studies we demonstrate that the levels of (SH)_2_Trx-ASK1 and the activity of downstream MKK3/6 and p38 MAPK correlate with levels of endogenous oxidative stress in *Klotho^(-/-)^* and *Klotho* overexpressing models. We propose that the mechanism of resistance to oxidative stress may involve the balance between the inASK1-signalosome *vs.* the actASK1-signalosome; that this mediates the level of activities of the p38 MAPK and SAPK/JNK pathways, and is a basic difference between wild-type and long-lived mice. To test our hypothesis, we measured (SH)_2_Trx-ASK1 complex levels, the activities of the components of the p38 MAPK and SAPK/JNK pathways and Nrf2 whose targeted genes promote resistances to oxidative stress, in the livers from *Klotho^(-/-)^* that exhibit elevated oxidative stress and in the *Klotho* overexpressing model that exhibits resistance to oxidative stress.

## RESULTS

### *Klotho^(-/-)^* exhibits low levels of the inhibitory (SH)_2_Trx-ASK1 complex

**Figure 1.**
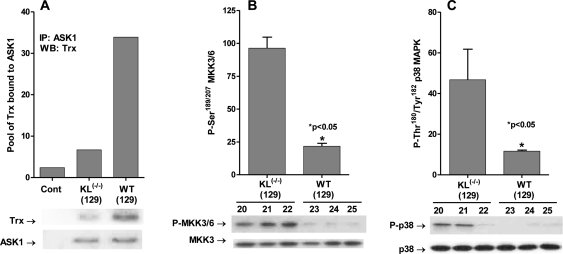
*Klotho* ablation activates the ASK1-signalosome - p38 MAPK pathway. (**A**) *Klotho^(-/-)^* exhibits low levels of the inhibitory (SH)_2_Trx-ASK1 complex. Western blot analysis of levels of Trx co-immunoprecipitated with ASK1 (IP: ASK1) in liver extracts of *Klotho^(-/-)^* (129) and WT (129) mice. (**B**) MKK3/MKK6 activity is elevated in *Klotho^(-/-)^*. Western blot analysis of levels of MKK3/6 catalytic site amino acids, P-Ser189/207 in livers of Klotho-/- and WT 129 mice. (**C**) p38 activity is elevated in *Klotho^(-/-)^*. Western blot analyses of levels of the p38 MAPK catalytic site amino acids, P-Thr180/Tyr182, in livers of Klotho (-/-) (129) and WT (129) mice. The bar graphs depict the mean +/- SE of samples from *Klotho^(-/-)^* (129; #20, 21, 22) and from WT (129; #23, 24, 25).

The *Klotho* gene encodes a single-pass transmembrane protein detected mainly in distal convoluted tubules of the kidney and choroid plexus of the brain [54]. Overexpression of the gene extends lifespan by ~20-30% in males and 18-19% in females, whereas ablation of the gene [*Klotho^(-/-)^*] leads to premature aging and death at ~2 months of age. It has been reported that the *Klotho* gene product plays a key role in the regulation of endogenous ROS [47]. Thus, the early death of *Klotho^(-/-)^* is attributed to increased levels of endogenous oxidative stress, whereas its extended lifespan seen in the *Klotho* overexpressing mouse is attributed to decreased levels of endogenous ROS. Since ROS levels correlate with longevity, we propose that part of the mechanism of regulation of longevity in the *Klotho* models may be linked to the regulation of the ASK1-signalosome → p38 MAPK activity. To test our hypothesis we conducted experiments to measure the levels of the inhibitory (SH)_2_Trx-ASK1 complex in wild-type (WT129) and *Klotho^(-/-)^* (129) mice. The data in Figure 1A clearly show a significant loss of the inhibitory (SH)_2_Trx-ASK1 complex in *Klotho^(-/-)^* as indicated by the amount of Trx pulled down by anti-ASK1 antibody.

### MKK3/6 activity is elevated in *Klotho^(-/-)^*

The MKK3/6 serine-threonine kinases are direct downstream substrates of the actASK1-signalosome. The data in Figure 1B show that phosphorylation of the catalytic site amino acids, Ser^189^ and Ser^207^, increased by ~4.5-fold in *Klotho^(-/-)^*. These data suggest that activation of the ASK1-Signalosome by increased oxidative stress in *Klotho^(-/-)^* activates the MKK3/6 serine kinases.

### p38 MAPK activity is elevated in *Klotho^(-/-)^*

Our hypothesis proposes that the increased levels of endogenous oxidative stress in *Klotho^(-/-)^* would result in increased activity of p38 MAPK via the activation of ASK1. The data in Figure 1C clearly show that phosphorylation of the catalytic site amino acids, Thr^180^ and Tyr^182^ is increased by ~4-fold in *Klotho^(-/-)^* livers. These data are consistent with our proposal that the ROS-mediated activation of p38 MAPK may occur via the dissociation of the inhibitory (SH)_2_Trx-ASK1 complex thereby activating the ASK1 → MKK3/6 → p38 MAPK pathway. Furthermore, the data support our proposal that this may be the pathway that sustains elevated p38 MAPK activity and activation of p38 MAPK targeted genes whose products promote the aging physiological phenotype.

### *Klotho* overexpression mediates increased levels of the inhibitory (SH)_2_Trx-ASK1 complex

*Klotho* has been shown to play a key role in the regulation of endogenous oxidative stress [47]. The data presented above suggest that ablation of the *Klotho* gene activates the ROS-sensitive ASK1-signalosome which then activates the downstream p38 MAPK stress response pathway. On the other hand, the consequences of *Klotho* overexpression, *i.e.,* the higher SOD2 expression and less phosphorylated FOXO [15] correlate *Klotho* activity with the development of resistance to oxidative stress. These data suggest that *Klotho* may regulate ROS-responsive signaling pathways that promote aging and longevity.

**Figure 2.**
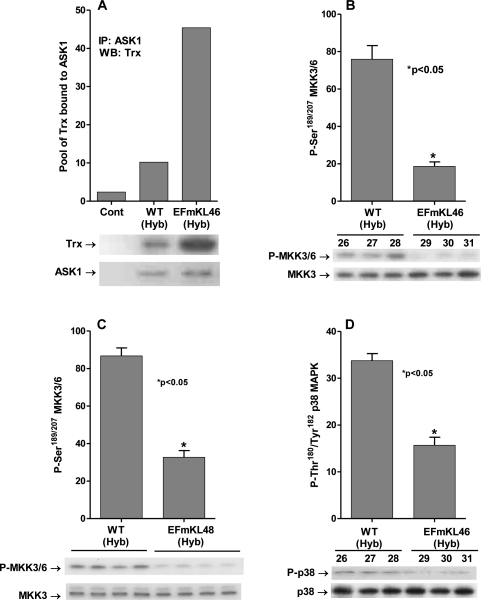
*Klotho* overexpression attenuates the ASK1-signalosome - p38 pathway. (**A**) Overexpression of *Klotho* mediates increased levels of the inhibitory (SH)_2_Trx-ASK1 complex. Western blot analysis of the levels of Trx co-immunoprecipitated with anti-ASK1 antibody in liver extracts of *Klotho* overexpressing (EFmKL46) and wild-type (WT Hyb) mice. (**B, C**) MKK3/MKK6 activity is downregulated in the *Klotho* overexpressing models. Western blot analysis of levels of the MKK3/6 P-Ser^189/207^ in livers of (**B**) *Klotho* overexpressing EFmKL46 (#29, 30, 31) and wild-type (WT Hyb; #26, 27, 28) mice and (**C**) *Klotho* overexpressing EFmKL48 (#652, 653, 654, 655); and (D) p38 MAPK activity is downregulated by *Klotho* overexpression. Western blot analysis of levels of p38 MAPK catalytic site amino acids (P-Thr^180^/Tyr^182^ ) in livers of WT Hyb (#26,27,28) *Klotho* over-expressing mice (EFmKL46 ; #29,30,31).

**Figure 3.**
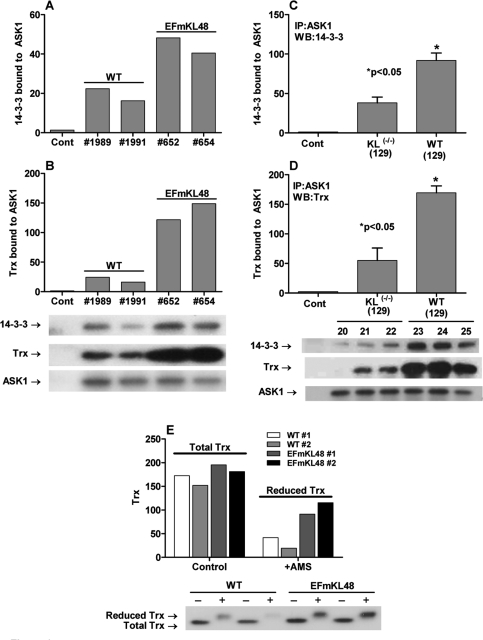
Binding of 14-3-3 and Trx to ASK1 is part of the inhibitory ASK1-signalosome complex. Assembly of the inhibitory ASK1-signalosome involves the binding of ASK1 to 14-3-3 [56]. Using anti-ASK1 for co-immunoprecipitation analyses we demonstrated (**A, C**) 14-3-3 and (**B, D**) Trx are complexed with ASK1. Furthermore, the data show that the ASK1-Trx-14-3-3 complex, a characteristic of the inhibitory ASK1-signalosome, is significantly elevated in the Klotho overexpressing model. (**E**) Treatment of the liver extracts with the thiol-reacting reagent shows a higher level of reduced Trx in the Klotho overexpressing liver.

Our previous studies demonstrated that dissociation of the inhibitory (SH)_2_Trx-ASK1 complex is stimulated by mitochondrial dysfunction due to rotenone-mediated inhibition of CI [21]. In addition we demonstrated that the levels of the complex are significantly decreased in aged (24-mo) compared to young (3-4-mo) C57BL/6 mice and that the levels of the inhibitory complex are significantly higher in livers of young (3-4 mo) and aged (24 mo) *Snell* dwarf mice at all ages. Furthermore, we demonstrated that the inhibitory complex is significantly higher in dermal fibroblasts derived from young (3-4 mo), middle aged (12-14 mo) and aged (20-24 mo) compared to fibroblasts derived from wild-type mice of the same ages and genetic background [31]. These data support our hypothesis that the ASK1-signalosome may serve as a ROS sensory distribution center that communicates changes in endogenous ROS levels to signaling pathways regulating physiological characteristics of aging. Based on these studies we propose that the levels of the inhibitory (SH)_2_Trx-ASK1 complex are indicative of the levels of endogenous oxidative stress. Thus, since the *Klotho* gene regulates endogenous oxidative stress we conducted experiments to determine whether the overexpression of *Klotho* would alter the pool levels of this inhibitory complex. To conduct these experiments we used two independent transgenic mouse lines, EFmKL46 and EFmKL48 that overexpress *Klotho* under the control of the human elongation factor 1α promoter. The data in Figure 2A clearly show that the level of the inhibitory (SH)_2_Trx-ASK1 complex is significantly elevated (~4.5-fold) in the EFmKL46 and ~6-fold in the EFmKL48 overexpressing models (Figure 3B). These data are consistent with our results showing that the complex levels in *Klotho^(-/-)^* are decreased and support our hypothesis that the complex may serve as a major ROS-sensitive center of distribution of signals of oxidative stress.

### MKK3/MKK6 activity is down regulated in the *Klotho* overexpressing model

Our hypothesis postulates that the decreased levels of endogenous oxidative stress in the *Klotho* overexpressing model would decrease the activity of the ASK1-signalosome and the signaling components of the p38 MAPK pathway. The data in Figure 2B and 2C show that the phosphorylation of MKK3/MKK6 at Ser^189^ and Ser^207^ is significantly down regulated in both the EFmKL46 (~3-fold) and EFmKL48 (~2.5-fold) models. These data are consistent with our hypothesis that *Klotho* mediated decrease of endogenous oxidative stress down regulates the p38 MAPK activity by attenuating the upstream MKK3/6 kinases.

### p38 MAPK activity is down regulated by *Klotho* overexpression

The decreased MKK3/MKK6 activity in the *Klotho* overexpressing models suggests that their down stream target, p38 MAPK should also be down regulated. The data in Figure 2D show that phosphorylation of the p38 MAPK catalytic site amino acids, Thr^180^ - Tyr^182^, is down regulated by ~2-fold in the *Klotho* overexpressing (EFmKL46) mouse liver. We thus propose that the ASK1-signalosome serves as an ROS-sensitive center for the distribution of signals of oxidative stress and the regulation of the level of p38 MAPK activity.

### Binding of 14-3-3 and Trx to ASK1 is part of the inhibitory ASK1-Signalosome complex

Sequestration of the inASK1-signalosome involves the binding of ASK1 to 14-3-3ζ[56]. Thus, signalosome activation in response to oxidative stress involves its release from 14-3-3. We propose, therefore, that the level of the sequestered ASK1-14-3-3 complex should be elevated in the *Klotho* overexpressing mutant. Using anti-ASK1 for co-immunoprecipitation analyses we demonstrated that both 14-3-3 and Trx are complexed with ASK1 (Figure 3A, B). Alternatively, we propose that the level of the sequestered ASK1-14-3-3 complex should be decreased in *Klotho^(-/-)^*. The co-immunoprecipitation data in Figure 3C and 3D clearly show that the ASK1-14-3-3 complex is significantly lower in the *Klotho^(-/-)^* mutant compared to its age-matched controls. The data thus show that the levels of the ASK1-Trx-14-3-3 complex, a characteristic of the inASK1-signalosome correlate with levels of oxidative stress in the *Klotho* models.

**Figure 7.**
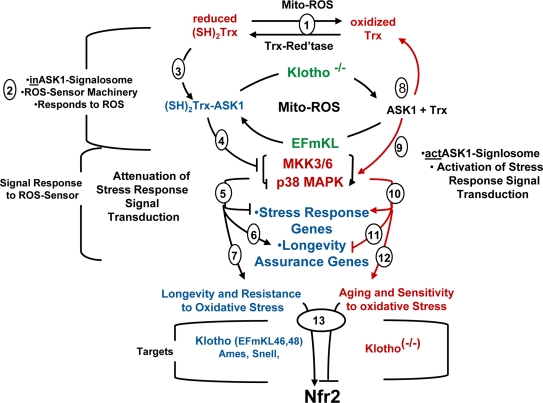
The oxidative stress-Chronic stress cycle of aging. Integration of the Role of the ASK1-Signalosome in ROS-Mediated Regulation of the p38 MAPK Pathway and Physiological Characteristics of Aging. [1] ROS generated by mitochondrial dysfunction; [2] the ASK1-signalosome responds to changes in levels of oxidative stress (ROS); [3] (SH)_2_Trx complexes with ASK1 to form (SH)_2_Trx-ASK1 complex, a component of the inASK1-signalosome; [4] the (SH)_2_Trx-ASK1 complex (inASK1-siganlosome) inhibits p38 MAPK activity; (5 → 7) inhibition of p38 MAPK activity attenuates stress response gene expression and favors expression of longevity assurance genes. This is the predominating pathway of the long-lived *Klotho* overexpressing, *Snell* and *Ames* mice that favors resistance to oxidative stress. [8] *Klotho* ablation causes increased endogenous ROS, dissociation of the (SH)_2_Trx-ASK1 complex to form the actASK1-signalosome. [9] ASK1 activates the p38 MAPK pathway and [10] p38 targeted genes that promote aging. (8 → 12) This is the predominant pathway of *Klotho ^(-/-)^* that promotes accelerated aging and sensitivity to oxidative stress; [13] The activation and nuclear localization of Nrf2 in the *Klotho* overexpressing mice and decreased Nrf2 activity in *Klotho^(-/-)^*^.^

To further support our model (Figure 7) that elevated levels of (SH)_2_Trx would correlate with the attenuation of the inASK-signalosome → p38 activity we measured the levels of total Trx *vs.* (SH)_2_Trx in the *Klotho* overexpressing model. The data in Figure 3E show that total Trx levels are similar in WT and EFmKL48 mice whereas the level of (SH)_2_Trx is significantly higher in the overexpressing model (Figure 3E). These results are consistent with the observation that the level of (SH)_2_Trx bound to ASK1-14-3-3 complex is significantly elevated in the EFmKL48.

### Levels of nuclear Nrf2 are altered in *Klotho^(-/-)^* and in the *Klotho* overexpressing mice

**Figure 4.**
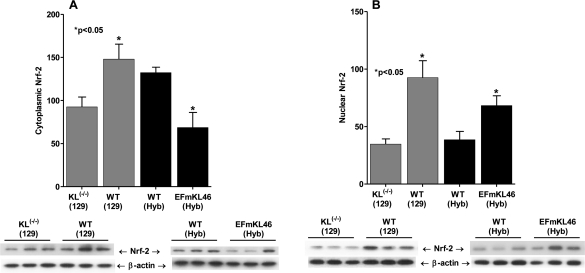
The effects of *Klotho^(-/-)^* and *Klotho* overexpression on the nuclear and cytoplasmic localization of Nrf2. (**A**) Cytoplasmic and (**B**) nuclear levels of Nrf2 in the KL^(-/-)^ and EFmKL46 Klotho overexpressing mice.

Nrf2 is a cytoplasmically localized transcription factor that, in response to oxidative stress, translocates to the nucleus where it controls the expression and coordinated induction of a battery of genes critical for cellular protection and survival. This mechanism involves the binding of Nrf2 to genes whose promoters carry the antioxidant response element (ARE; [57,58]. Thus, the ARE genes contribute to the physiological defense against oxidative stress and to the development of resistance to oxidative stress [59,60]. We therefore, propose that intracellular localization of Nrf2 would respond to the increased oxidative stress in *Klotho^(-/-)^* and decreased oxidative stress in *Klotho* overexpressors. Interestingly, the data in Figure 4A and 4B show a significant decrease of levels of both cytoplasmic and nuclear-localized Nrf2 in *Klotho^(-/-)^* compared to WT suggesting that this mutant may exhibit a decreased level of the protective activity of Nrf2 targeted genes and therefore, may explain its accelerated aging. On the other hand, the overexpression of *Klotho* results in significantly increased levels of Nrf2 sequestered in the WT cytoplasm compared to nuclear levels. Furthermore the nuclear localization of Nrf2 in EFmLK 46 increase while the cytoplasmic level decreases suggesting activation of the anti-oxidant ARE targeted genes. These data are consistent with the development of resistance to oxidative stress in the *Klotho* overexpressing model and suggest that activation of Nrf2 may be an as yet uncharacterized function of *Klotho*.

### ASK1 pool levels are not altered in *Klotho^(-/-)^* in *Klotho* overexpressing mice

**Figure 5.**
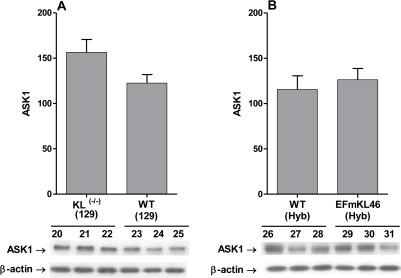
Western blot analysis of pool level of ASK1 in the livers of *Klotho^(-/-)^* and *Klotho* overexpressing mice. Western blot analyses of levels of ASK1 in the livers of (**A**) *Klotho^(-/-)^*(129)and WT (129) mice and (**B**) the EFmKL46 *Klotho* overexpressing and WT mice. The bar graphs depict the mean +/- SE of samples from WT, *Klotho^(-/-)^* and EFmKL 46 *Klotho* overexpressing mice.

The data in Figure 5A and 5B show that there is no change in the ASK1 protein pool levels in *Koltho^(-/-)^* or the *Klotho* overexpressing models compared to their wild type controls. We conclude that the responses to *Klotho* ablation or overexpression involve post-translational modifications of the ASK1-signalosome.

### The p46 SAPK/JNK isoform is activated in *Klotho^(-/-)^*

**Figure 6.**
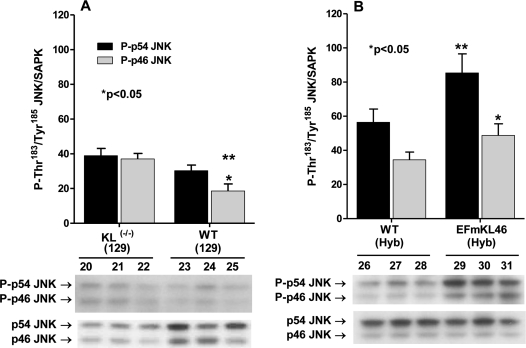
Western blot analysis of the phosphorylation of the p46- and p54-JNK. The bar graphs and immuno blots show levels of phosphorylation of the catalytic site amino acids, P-Thr^183^/Tyr^185^, of p46- and p54-JNK in livers of *Klotho ^(-/-)^* (129) and WT (129) mice; and (**B**) in EFmKL 46 *Klotho* overexpressing and WT mice.

The SAPK/JNK signaling processes involve activation of the p46-JNK and p54-JNK isoforms. The ASK1-signalosome activates these signaling proteins via MKK4/MKK7 kinases [27]. However, the p46-JNK and p54-JNK isoforms are differentially activated in response to certain challenges. For example, the preferential activation of p54 by 3-NPA, an inhibitor of succinic dehydrogenase (ETC CII), in young C57BL/6 mouse livers is altered in aged mice [19]. The data in Figure 6A show that in WT (129) phosphorylation of Thr^183^ / Tyr^185^ in p46-JNK is lower than in p54-JNK and that only the phosphorylation of p46-JNK increases significantly in *Klotho^(-/-)^.* Thus, the data suggest a specific activation of p46-JNK by increased oxidative stress due to *Klotho* ablation and that the genes targeted by p46-JNK may play a role in accelerated aging of *Klotho^(-/-)^*.

### SAPK/JNK isoform activities increase in *Klotho* overexpressing mice

The data in Figure 6B show that the responses of p46-JNK and p54-JNK to *Klotho* overexpression are similar, *i.e.,* ~2-fold increase for P-p46-JNK and ~1.7-fold increase for P-p54-JNK. Interestingly, the data also show that *Klotho* ablation (Figure 6A) and *Klotho* overexpression (Figure 6B) exhibit the same response, *i.e.,* phosphorylation of the p46-JNK catalytic site amino acids. Thus, both *Klotho* ablation and *Klotho* overexpression activate p46- and p54-JNK (Figure 6A, 6B). These data suggest that the physiological responses to *Klotho* ablation and overexpression may activate specific signaling processes unique to *Klotho* function and raises the question of whether genes targeted by these differential responses play a role in longevity determination.

## DISCUSSION

The increase in ROS and its promotion of physiological processes of aging emphasizes the importance of understanding the molecular mechanism of ROS-linked modulation of aging, longevity and resistance to oxidative stress [11]. It is well recognized, for example, that increased p38 MAPK activity plays a role in the activation of senescence and aging phenotypes [16,18-21]. Furthermore, mouse models exhibiting extended lifespan, *i.e.,Klotho, Snell* and *Ames* dwarf mice share characteristics of low levels of endogenous oxidative stress and resistance to oxidative stress [14,15,45,49,50]. Thus, increased endogenous ROS levels and p38 MAPK activity are recognized as major signaling factors that promote senescence (*in vitro*), aging and longevity (*in vivo*) [7-10,19]. In this study we demonstrate that the ROS-sensitive ASK1-signalosome is a modulator of p38 MAPK activities in the age-accelerated *Klotho^(-/-)^* model and in the *Klotho* overexpressing model of extended lifespan. Our results suggest that the increased ROS generated by *Klotho^(-/-)^*, and resistance to oxidative stress in *Klotho* overexpression may modulate ROS-sensitive ASK1-signalosome activity [21]. We present evidence that increased oxidative stress in *Klotho^(-/-)^* activates the ASK1-signalosome → p38 pathway which may be an important physiological factor that accelerates aging characteristics of this mutant [15]. Furthermore, we propose that the mechanism of this activation involves the dissociation of the inhibitory (SH)_2_Trx-ASK1 complex which converts the inASK1-signalosome to the actASK1-signalosome thereby identifying a link between elevated ROS, activation of p38 MAPK and the accelerated aging of *Klotho^(-/-)^*. Although we have not shown directly that ROS induced by *Klotho^(-/-)^* is due to mitochondrial ETC dysfunction, our studies suggest that the ability of *Klotho* to function as a suppressor of aging may include the protective role of the ROS-sensitive inASK1-signalosome as well as increased MnSOD activity. The fact that overexpression of *Klotho* decreases the activity of the ASK1-signalosome → p38 MAPK suggests that suppression of aging associated with lower levels of oxidative stress may be due to the attenuation of signaling pathways targeted by p38 MAPK as well as to higher SOD2 expression and decreased levels of FOXO phosphorylation [15]. Our studies suggest that the ASK1-signalosome serves as an ROS-sensory system that regulates the activity of the ROS-responsive physiological signaling processes that link ROS levels to the modulation of aging characteristics. Interestingly, *Klotho^(-/-)^* shows an increase in apoptosis [14]. Thus, since the ASK1-signalosome also serves as a regulator of mitochondrial-mediated apoptosis the attenuation of this activity by *Klotho* overexpression further supports our hypothesis that this ROS-sensitive complex plays an important role in *Klotho's* function as a suppressor of aging [47,49]. We thus suggest that the increased apoptosis in *Koltho^(-/-)^* may be mediated by increased actASK1-signalosome activity.

Since mitochondrial DNA damage is also a source of ROS due to the production of 8-oxoG, the ability of the *Klotho* overexpressing transgenic mouse to decrease circulating levels of 8-oxoG, and its activation of mitochondrial MnSOD suggests that these *Klotho* activities may be a part of the mechanism of resistance to oxidative stress [15].

The significantly higher levels of the inhibitory (SH)_2_Trx-ASK1 complex in *Klotho* overexpressing mice and the decreased levels of this complex in *Klotho^-/-^* provides genetic evidence for the importance of the level of endogenous oxidative stress in determination of their longevity. We propose that the level of (SH)_2_Trx-ASK1 (inASK1-signalosome) may be a physiological contributor to the *Klotho*-mediated regulation of aging or longevity. This hypothesis is strongly supported by our studies showing that in the *Klotho* overexpressing mice the levels of the inhibitory (SH)_2_Trx-ASK1 complex are significantly higher whereas in *Klotho ^(-/-)^* the levels are decreased. We conclude that in the *Klotho, Ames* and *Snell* mice the physiological functions of the ROS-sensitive ASK1-signalosome play a role in the determination of their extended lifespan and resistance to oxidative stress. Our proposal that the level of the (SH)_2_Trx-ASK1 complex in the inASK1-signalosome is indicative of and part of the mechanism of resistance to oxidative stress is supported by the fact that the dissociation of the complex thus forming the activated signalosome is much more severe in the *Klotho^(-/-)^* and that the formation of the inASK1-signalosome is significantly elevated in the oxidatively resistant *Klotho* overexpressing model. Further support of our hypothesis stems from the observation that the levels of (SH)_2_Trx in the total Trx pool and that nuclear localization of the Nrf2 transcription factor are significantly higher in the *Klotho* overexpressing model. In fact we further propose that the decreased levels of nuclear Nrf2 in *Klotho^(-/-)^* may account for its accelerated aging due to decreased physiological protection.

The mechanism by which *Klotho* increases resistance to oxidative stress involves its promotion of nuclear localization of FOXO thereby stimulating SOD2 expression [14]. Our studies show that *Klotho* also stimulates the nuclear translocation of Nrf2 adding the multiple anti-oxidant genes regulated by the ARE to *Klotho*'s function that confers resistance to oxidative stress.

Our past studies have shown that aging tissues develop a state of chronic stress as indicated by the increased basal levels of the p38 MAPK and SAPK/JNK signaling activities [4,16,19]. Our present studies suggest that the dissociation of the (SH)_2_Trx-ASK1 complex is promoted by elevated endogenous ROS levels due to *Klotho* ablation, thereby sustaining the p38 MAPK activity and its targeted aging and senescence processes. Our studies have indeed shown that the levels of oxidized vs reduced Trx are affected by the ablation or overexpression of *Klotho* and thus suggest that the activity of thioredoxin reductase may also be regulated by *Klotho.* We propose (a) in *Klotho^(-/-)^* the ratio of the actASK1-signalosome : inASK1-signalosome shifts toward the dissociation of the (SH)_2_Trx-ASK1 complex, which activates ASK1 → p38 MAPK; (b) in *Klotho* overexpression the ratio shifts toward the association of the complex which attenuates ASK1 → p38 MAPK activity (Figure 7); (c) this is the mechanism for increased and sustained activity of p38 MAPK in *Klotho^(-/-)^* and in aged tissues, and the physiological mechanism of development of aging characteristics [24]. (d) The severe loss of resistance to oxidative stress which is strongly suggested by decreased level of nuclear localized Nrf2, would explain the extreme and rapid loss of tissue integrity in *Klotho^(-/-)^*.

Our hypothesis is also supported by certain characteristics of the oxidative stress resistant *Snell* and *Ames* dwarf mutants. This includes the increased levels of the inhibitory (SH)_2_Trx-ASK1 complex which accounts for the decreased activity of the components of the p38 MAPK pathway, MKK3 kinase activity, nuclear P-p38 kinase and ATF-2 and which is associated with the resistance to oxidative stress in both young and aged dwarf mice [21]. Furthermore, the significantly higher level of reduced thioredoxin in dwarf cells is consistent with the higher (SH)_2_Trx-ASK1 levels and resistance to oxidative stress.

Interestingly, since p38 MAPK is a master regulator of many genes, our proposed mechanism implies that the ASK1-signalosome-mediated regulation of p38 MAPK activity should affect multiple genetic responses associated with aging determination and resistance to oxidative stress. This is consistent with the observation that ASK1 is selectively required for sustained activation of the p38 MAPK (and SAPK/JNK) pathways induced by oxidative stress [53]. This was demonstrated in ASK1^(-/-)^ embryonic fibroblasts in which the H_2_O_2_ and TNF-mediated sustained activation of p38 MAPK and SAPK/JNK is lost. These cells also exhibit elevated resistance to oxidative stress. Thus the elevated level of the inhibitory complex and attenuation of ASK1 → p38MAPK activity in *Ames* fibroblasts mimics the resistance to oxidative stress shown by the ASK1^(-/-)^ embryonic fibroblasts. Furthermore, the elevated inASK1-signalosome levels and attenuated p38 MAPK activity in livers of *Snell* dwarf mice [21] suggests that this may contribute to their resistance to oxidative stress. At the same time, the constitutively elevated and sustained p38 MAPK activity in the livers of aged wild-type mice may be due to the increased pool level of the activating signalosome. Our hypothesis predicts that treatment of aged wild-type and *Klotho^(-/-)^* mice with anti-oxidants may reverse the elevated endogenous levels of p38 MAPK activity, increase their resistance to oxidative stress and possibly increase their lifespan.

Resistance to oxidative stress is an important physiological factor in longevity [50]. Our results suggest that attenuation of the p38 MAPK pathway in *Klotho* overexpressing mice down regulates its targeted senescence/aging pathways and may, therefore, be the physiological basis for their resistance to oxidative stress. The decreased state of oxidative stress in *Klotho* overexpressing mice and in young and aged *Snell* and *Ames* mice correlates with their decreased levels of p38 MAPK activity and their resistance to oxidative stress [16,19]. Thus, the lower level of endogenous oxidative stress in these longevity models attenuates the ASK1-signalosome →p38 MAPK activity which favors longevity.

Our studies raise the question of whether the elevated (SH)_2_Trx-ASK1 levels are part of the mechanism of resistance to oxidative stress. For example, activation of p38 MAPK in ASK1^(-/-)^ embryonic fibroblasts by H_2_O_2_ and TNF is abolished in these cells which are resistant to H_2_O_2_- and TNF-induced apoptosis [53]. ASK1 activity is, therefore, required for the sustained activation of p38 MAPK by these ROS generating factors. Thus, the regulation of levels of reduced thioredoxin and the (SH)_2_Trx-ASK1 complex may be part of the mechanism of resistance to oxidative stress in long-lived mice [50].

We have shown that the levels of phosphorylation of p46-JNK and p54-JNK are increased in the *Klotho* overexpression livers. This is a unique physiological response that may not be linked to the ASK1-mediated activation of SAPK/JNK, since the inhibitory (SH)_2_Trx-ASK1 complex is elevated in this model. Thus, it appears that the *Klotho* activation of p46-JNK and p54-JNK may be mediated via an as yet unidentified signaling pathway that is linked to *Klotho* expression. Our data suggest, therefore that the activation of SAPK/JNK in *Klotho^-/-^* may be mediated via the stress response ASK1 activation associated with aging and senescence whereas the activation in response to *Klotho* overexpression is not a stress response and may, therefore, be part of the longevity characteristics regulated by the circulating *Klotho* hormone.

Trx can exert its protective functions either directly as an antioxidant or indirectly by binding to signaling components and modulating their functions. The model we present in this study proposes that complexing of (SH)_2_Trx with the N-terminus of ASK1 modulates the ROS mediated activity of the p38 MAPK pathway (Figure 7) The elevated levels of reduced Trx and Trx bound to ASK1 in the *Klotho* overexpression models, in young and aged *Ames* fibroblasts and in *Snell* dwarf livers [21] suggest that the tissues of these long-lived mice are in a more reduced state than their age-matched controls, and that they maintain a lower level of p38 MAPK activity throughout their life cycle supports the hypothesis that their endogenous redox state favors the attenuation of physiological aging. Thus, our model proposes that the phenotypes of longevity and resistance to oxidative stress of the *Klotho* overexpression and dwarf mice involves the down regulation of the stress response pathway and stress response genes associated with the development of aging characteristics and that this is a function of the ROS-sensory machinery i.e., the ASK1-signalosome that serves as a distribution center for the dissemination of ROS signals to responsive signaling pathways (Figure 7). Alternatively, the aging phenotypes of *Klotho^-/-^* and other models of increased oxidative stress involves elevated levels of the actASK1-siganlosome thereby sustaining the elevated levels of stress response signaling processes that promote the development of physiological characteristics of aging. Our studies provide further support of the hypothesis that the age-associated increase in endogenous ROS may be a major factor in the sustained increase in stress response activity in aged tissue via the ASK1-signalosome ROS signaling distribution center thereby modulating the development of age-associated physiological characteristics.

## METHODS

Animals and tissues. Klotho-deficient mice and Klotho-overexpressing transgenic mice were described previously [47,54]. The original Klotho-deficient mice were backcrossed to 129Sv inbred mice for more than 9 generations to establish a 129Sv congenic Klotho-deficient mouse line. Wild-type 129Sv inbred mice were used as a control for the Klotho-deficient mice. Two independent transgenic mouse lines that overexpress Klotho, EFmKL46 and EFmKL48, has a hybrid genetic background (87.5% C3H and 12.5% C57BL/6). Wild-type mice with the same genetic background were used as controls for these transgenic mice. Mice were placed on a standard rodent chow and housed in the same room under specific-pathogen free conditions. Tissues were harvested from males at 8 weeks of age, frozen immediately in liquid nitrogen, and stored at -80°C until they were used for protein extraction. All animal experiments were approved by the Institutional Animal Care and Use Committee of the University of Texas Southwestern Medical Center at Dallas.

### Preparation of *Klotho* liver extracts

Liver extracts were prepared as described previously [16,61,62] with some modifications. After centrifugation to collect the cytosolic fraction, the pellet was extracted as described [16]. The concentrations of the cytosolic and nuclear samples were determined using Bradford assay reagent (Bio-Rad).

### Western blot and immunoprecipitation assays

Western blot and immunoprecipitation assays were performed as described [16]. The antibodies against SAPK/JNK (#9252) and MKKs (#9232), and the phosphor-antibodies (P-p38 MAPK (#9215), P-MKK3/6 (#9236), P-SAPK/JNK (#4668) were purchased from Cell Signaling Technology. The ASK1 (SC-7931), p38 MAPK (SC-535), Trx (SC-20146), 14-3-3 (SC-629) antibodies were obtained from Santa Cruz Biotechnology. The protein A-conjugated agarose beads for immunoprecipitation assay and β-actin (A1987) antibody were obtained from Sigma. Preimmune rabbit serum was used to react with wild-type extract in immunoprecipitation assays as a control to show the specific binding of thioredoxin and 14-3-3 to ASK1 antibody.

### Determination of levels of reduced thioredoxin

The procedure for determination of the endogenous levels of reduced thioredoxin has been described [31].

### Statistical analysis

Data are presented as means ±SE. Statistical analyses were performed using unpaired Student's *t* test to determine the statistically significant differences between wild-type *vs.Klotho*^(-/-)^mouse livers or wild-type vs. *Klotho* overexpressing mouse livers. A *p* value less than 0.05 was considered as statistically significant.
